# A pragmatic method for costing implementation strategies using time-driven activity-based costing

**DOI:** 10.1186/s13012-020-00993-1

**Published:** 2020-05-05

**Authors:** Zuleyha Cidav, David Mandell, Jeffrey Pyne, Rinad Beidas, Geoffrey Curran, Steven Marcus

**Affiliations:** 1 Department of Psychiatry, Perelman School of Medicine, University of Pennsylvania, Philadelphia, PA USA; 2 Leonard Davis Institute of Health Economics, University of Pennsylvania, Philadelphia, PA USA; 3 Center for Mental Healthcare and Outcomes Research, Central Arkansas Veterans Healthcare System, North Little Rock, AR USA; 4 South Central Mental Illness Research, Education and Clinical Center, Central Arkansas, Little Rock, USA; 5 Veterans Healthcare System, North Little Rock, AR USA; 6 Division of Health Services Research, Department of Psychiatry, College of Medicine, University of Arkansas for Medical Sciences, Little Rock, AR USA; 7 Department of Medical Ethics and Health Policy, Perelman School of Medicine, University of Pennsylvania, Philadelphia, PA USA; 8 Departments of Pharmacy Practice and Psychiatry, University of Arkansas for Medical Sciences, Little Rock, AR USA; 9 Center for Implementation Research, University of Arkansas for Medical Sciences, Little Rock, AR USA; 10 Central Arkansas Veterans Healthcare System, Little Rock, AR USA; 11 School of Social Policy and Practice, University of Pennsylvania, Philadelphia, PA USA; 12 Department of Medicine, Perelman School of Medicine, University of Pennsylvania, Philadelphia, PA USA; 13 Penn Implementation Science Center, Leonard Davis Institute of Health Economics, Philadelphia, USA

**Keywords:** Costing, Economic evaluation, Implementation strategies, Time-driven activity-based costing

## Abstract

**Background:**

Implementation strategies increase the adoption of evidence-based practices, but they require resources. Although information about implementation costs is critical for decision-makers with budget constraints, cost information is not typically reported in the literature. This is at least partly due to a need for clearly defined, standardized costing methods that can be integrated into implementation effectiveness evaluation efforts.

**Methods:**

We present a pragmatic approach to systematically estimating detailed, specific resource use and costs of implementation strategies that combine time-driven activity-based costing (TDABC), a business accounting method based on process mapping and known for its practicality, with a leading implementation science framework developed by Proctor and colleagues, which guides specification and reporting of implementation strategies. We illustrate the application of this method using a case study with synthetic data.

**Results:**

This step-by-step method produces a clear map of the implementation process by specifying the names, actions, actors, and temporality of each implementation strategy; determining the frequency and duration of each action associated with individual strategies; and assigning a dollar value to the resources that each action consumes. The method provides transparent and granular cost estimation, allowing a cost comparison of different implementation strategies. The resulting data allow researchers and stakeholders to understand how specific components of an implementation strategy influence its overall cost.

**Conclusion:**

TDABC can serve as a pragmatic method for estimating resource use and costs associated with distinct implementation strategies and their individual components. Our use of the Proctor framework for the process mapping stage of the TDABC provides a way to incorporate cost estimation into implementation evaluation and may reduce the burden associated with economic evaluations in implementation science.

Contributions to the literature

- Implementation strategies help healthcare settings to adopt evidence-based practices, but stakeholders often cite their cost as a barrier. More detailed information about costs, including information that aids logistical decisions, could boost adoption.
- We apply an established method from the business accounting literature, time-driven activity-based costing, to implementation by combining it with the Proctor et al. framework, an established framework often used in implementation effectiveness evaluations to guide the specification and reporting of implementation strategies.
- This method can serve as a pragmatic tool to operationalize the conduct of implementation activities, determine resource consumption, and estimate associated costs. It could facilitate routine economic evaluations in implementation research.

## Background

The ability to assess the cost-effectiveness of evidence-based practices (EBPs) is critical to ongoing efforts to improve healthcare quality and reduce healthcare costs. This includes assessing the costs associated with implementation strategies or the activities required to achieve successful uptake of EBPs. Cost concerns are associated with reduced stakeholder willingness to implement EBPs, and thus, they have emerged as the most significant and least modifiable barrier to EBP implementation [1]. In particular, despite growing evidence that EBPs can be cost-effective over time, start-up costs are frequently cited as a major barrier to implementation [2, 3]. These costs may include external trainer and consultant time, internal staff time spent on training and receiving consultation, data collection and reporting, additional supervision, fidelity monitoring, and spending on travel, books, and other required materials.

In keeping with the fact that a clear understanding of implementation costs is important for determining the feasibility and achieving buy-in from stakeholders, many implementation frameworks highlight cost as an important factor to consider when evaluating implementation approaches and outcomes [3, 4]. However, there is little guidance with regard to how to pragmatically estimate these costs [5–7]. Few implementation science studies have considered costs, and even fewer have compared the costs of various implementation strategies, despite numerous calls for this type of research [8, 9]. For example, a systematic review of 235 implementation studies found that only 10% provided information about implementation costs [9]. In the rare cases where implementation costs are reported, usually they are presented as broad categories of spending (e.g., personnel, supplies, travel) [10, 11]. This approach uses total expenditure data (or program budget) to provide gross average estimates of costs. This information is helpful, but it does not offer sufficient detail to guide important decisions.

There have been several efforts to break down aggregate costs into more specific estimates of the resources consumed by various implementation activities. Recently, Ritchie et al. estimated the cost of facilitation as a strategy to implement integrated primary care and mental healthcare services [12]. They listed various activities performed by facilitators and stakeholders and regularly collected data on time spent on each activity to estimate costs. Similarly, the Cost of Implementing New Strategies (COINS) framework was designed to map costs onto pre-determined implementation stages and activity categories associated with the Stages of Implementation Completion (SIC) measure, an 8-stage tool to assess implementation process and milestones [13]. Liu et al. have broken down the implementation strategy of provider education into activities such as preparing and producing educational materials, providing introductory educational conferences, in-person or video conferencing for supervision and consultation, ongoing seminars, and academic detailing [14]. O’Beirne et al. broke down recruiting clinics into activities such as revising mail-out responses and preparing for and attending meetings [10].

We sought to build on these efforts by introducing a process-based approach for costing implementation strategies, borrowed from business settings, and mapping it on to an established implementation science framework that can be used to guide effectiveness evaluations. Time-driven activity-based costing (TDABC) is a micro-costing method that is widely used in business settings to determine various ways in which the structure of business activities can be redesigned to realize potential areas for improvement [15]. It does this by delivering more detailed, accurate, and transparent activity cost information that is more relevant and actionable for decision-making [10, 11, 16–18]. Its utility has led to its adoption by other sectors, including the service and healthcare industries [15, 19–22]. TDABC is well matched to implementation science’s focus on processes and procedures in the uptake of EBPs. The Proctor framework for identification, specification, and reporting of implementation strategies offers a guideline to be followed when evaluating the effectiveness of implementation strategies [23]. Our proposed method integrates TDABC and the Proctor framework and provides a step-by-step approach to costing implementation strategies. In this paper, we outline the method in detail and present a case study of its application.

## Method and case study

### Time-driven activity-based costing

TDABC is a process-based micro-costing methodology that provides detailed cost data through the use of process maps. It is particularly useful when costs are driven mainly by personnel time, hence the name “time-driven” [20]. Under the TDABC framework, a process is a chain of activities performed in combinations to achieve a certain purpose, such as delivering a service. Processes can be defined at any level of detail, starting at the most abstract and becoming more concrete by successive decomposition [24]. Specifically, each process can be broken down into a set of related procedures that describe the exact steps to complete the process.

The general steps of TDABC, adapted from Kaplan et al. [20], are (1) creation of a process map that outlines the procedures that comprise the process, where the procedures are defined as quantifiable events; (2) determination of the frequencies of those defined procedures; (3) estimation of the time-per-unit for the procedure, which specifies how long it takes to execute one unit of the procedure (one single event); (4) calculation of the total time spent to complete the procedure (i.e., multiplying the frequency of the procedure’s occurrence by the time-per-unit); (5) estimation of the cost per hour for each resource used; and (6) calculating the total cost of the procedure by multiplying the total procedure time (in hours) by the per-hour cost of resources. Summing the costs of procedures that comprise a process yields the process costs. In short, TDABC requires information on *who* (personnel completing the task) *does what* (specific activities performed), *when* (timing), and *how often* (the frequency, intensity and/or duration of the activity) [25].

TDABC is a modified version of activity-based costing (ABC), an earlier approach to cost accounting that relies on employee self-reported data to determine the percentage of time spent on the activities, or on ongoing activity time logs [20, 21]. Data generated in that manner can be time-consuming and costly to collect as well as difficult to validate [15, 19, 20]. TDABC changes how the time data are collected and modifies the calculation of activity costs [15, 20, 26, 27]. To do so, it requires only three parameters: (1) frequency of the activity (i.e., how many times the activity occurs), (2) time required to perform one single event of the activity (e.g., 1 hour), and (3) per-hour price of the resources (e.g., personnel time) used to perform the activity [16, 17]. This approach reduces the burden of time use data collection [20–22, 26] and the risk of inaccurate or subjective employee-reported data because the frequency and average duration of activities can typically be observed objectively (e.g., by checking the time stamp on a recorded consultation session) costs [15, 19–21, 26, 27], or they can be standardized and specified in advance in a way that does not require observation (e.g., a standard 3-h training session). TDABC has been shown to be practical when applied in various organizational settings including healthcare [15, 19, 22, 28]. Under TDABC, rough accuracy (i.e., how close your cost estimate is to the actual cost) is sufficient; precision (i.e., the number of decimal places included in the estimation) is not critical [20, 21]. In addition, because TDABC relies on granular, procedure-level costs as opposed to broad categories, it allows stakeholders to see what resources were used for what purposes. For example, it disaggregates the costs of an output (e.g., a consultation meeting) into the specific inputs or items consumed during its implementation (e.g., clinician time, travel costs, consumable supplies), which can help identify areas for cost savings.

### Proctor et al. framework for implementation strategy specification and reporting

Implementation strategies in existing literature have been criticized as being poorly described, inconsistently labeled, and insufficiently detailed [8, 23]. In response, many guidelines emerged that sought to develop a more standardized language for implementation strategies and other aspects of implementation [23, 29–31, 23, 29–32]. One set of guidelines, proposed by Proctor and colleagues [23], suggests that when studying implementation strategies, researchers should first name or label the implementation strategies in ways that are consistent with the published literature; then define them conceptually (i.e., give a general sense of what the strategy might involve); and finally, operationalize them by carefully specifying the following elements: (1) actor (i.e., who enacts the strategy?), (2) action(s) (i.e., what are the specific actions, steps, or processes that need to be enacted?), (3) action target (i.e., what constructs are targeted?), (4) temporality (i.e., when is the strategy used?), (5) dose (i.e., what is the intensity?), (6) implementation outcome (i.e., what implementation outcome(s) are likely to be affected by each strategy?), and (7) justification (i.e., what is the empirical, theoretical, or pragmatic justification for the choice of an implementation strategy?) [23, 33]. This approach has increasingly been applied in implementation research [6, 33, 34], and the Standards for Reporting Implementation Studies (STARI), a widely accepted checklist for transparent and accurate reporting of studies that evaluate implementation strategies [32], recommends this framework to describe implementation strategies.

### Costing implementation strategies using the TDABC and Proctor et al. framework

The information on implementation strategies generated by following the Proctor et al. framework aligns directly with the information needed for a TDABC approach to costing. This enables a novel application of the Proctor framework in assessing implementation strategy costs. In short, both approaches require specification of who, does what, when, how often, and for how long [23]. Following the Proctor et al. framework and generating information on actor (who), action (does what), temporality (when), and dosage (how often, for how long) allows it to guide application of the first step in the TDABC approach: process mapping to operationalize each implementation strategy to be used in a given project.

In our proposed method, we conceptualize an implementation strategy as a process associated with executing a series of specific procedures (actions) performed by utilizing human resources (actors) and non-human resources. Because the cost of an implementation strategy is primarily driven by the time spent by the actors executing its specific actions, for simplicity, we describe how our proposed approach would be used to estimate the cost of personnel resources, which is usually the largest time-driven cost item in implementation efforts. However, the estimation of the time-driven cost of non-personnel resources (e.g., office space rented monthly to perform a specific implementation strategy action) can be carried out similarly. If those costs are to be included, any non-personnel resources with time-driven costs would be listed for each action, alongside the actors.

Next, we use a case study and synthetic data to demonstrate how this step-by-step method can be used in implementation science.

## Results

### Case study: costing “General Practice Facilitation,” a multicomponent evidence-based implementation strategy

General practice facilitation is an evidence-based implementation strategy for assisting healthcare practices in implementing EBPs or improving in areas such as patient access and care [35]. Usually outside experts, practice facilitators, or practice coaches work closely with healthcare practices to develop the capacity for sustained change and improvement. In this case study, a primary care practice receives practice facilitation using multiple implementation strategies for the implementation of two psychotherapy EBPs. Using the adapted TDABC framework for costing implementation strategies and the Proctor et al. framework as a guide, we implement our method as outlined below. Appendix 1 presents a sample TDABC costing matrix that serves as a blueprint for this process, and Table 1 displays a completed matrix for our case study.

**Table 1:** Costing practice facilitation using the time-driven activity-based costing method

Implementation strategy	Actions	Temporality	Actors	Action frequency	Action unit duration (hours)	Total time spent on action (hours)	Actor wage rate ($)	Total cost ($)
I	II	III	IV	V	VI	VII	VIII	IX
Initial work for site readiness	Meet with partners in person	Pre-implementation, weekly for 3 months (i.e., 12 times) Action start and end date	1 general practice facilitator	12	2	24	50	1200
			1 site administrator	12	2	24	45	1080
			3 clinicians	12	2	72	55	3960
	Travel time to in-person meetings with partners	Pre-implementation, weekly for 3 months Action start and end date	1 general practice facilitator	12	1	12	50	600
	Communicate with partners via phone	Pre-implementation, weekly for 3 months Action start and end date	1 general practice facilitator	24	0.5	12	50	600
			1 site administrator	24	0.5	12	45	540
	Communicate with partners via email	Pre-implementation, weekly for 3 months Action start and end date	1 general practice facilitator	60	0.25	15	50	750
			1 site administrator	60	0.25	15	45	675
Initial training of partners	Meet with partners in person to deliver the training workshop	Pre-implementation, 2 full days Action start and end date	1 practice facilitator	2	8	16	50	800
			1 site administrator	2	8	16	45	720
			3 clinicians	2	8	48	55	2640
	Monitor training workshop via videoconferencing	Pre-implementation, 2 full days Action start and end date	1 expert consultant	2	8	16	65	1040
Ongoing administrative support	Meet with partners via videoconferencing for administrative issues	Implementation, biweekly for 12 months Action start and end date	1 practice facilitator	24	1	24	50	1200
			1 site administrator	24	1	24	45	1080
	Consult partner as needed by phone	Sustainment, for 6 months Action start and end date	1 practice facilitator	12	0.5	6	50	300
			1 site administrator	12	0.5	6	45	270
Remote consultation	Observe EBP delivery sessions and provide feedback via videoconferencing	Weekly first month of implementation, biweekly 2–4 months, monthly 5–8 months, bi-monthly 9–12 months Action start and end date	1 expert consultant	16	1	16	65	1040
			3 clinicians	16	1	48	55	2640
	Give ongoing clinical training via video conferencing	During implementation, as needed Action start and end date	1 expert consultant	10	1	10	65	650
			1 clinician at a given time	10	1	10	55	550
	Consult clinicians in scheduled group consultation sessions by phone	Implementation, biweekly for 12 months Action start and end date	1 expert consultant	24	1	24	65	1560
			3 clinicians	24	1	72	55	3960
	Consult clinicians individually as needed by phone	During implementation, for 12 months Action start and end date	1 expert consultant	48	0.5	24	65	1560
			1 clinician at a given time	48	0.5	24	55	1320
Fidelity review	Watch recorded EBP delivery sessions and fill out fidelity assessment forms	During implementation, monthly Action start and end date	1 expert consultant	36	1	36	65	2340
		Sustainment, 6 months, bi-monthly Action start and end date	2 expert consultants	9	1	9	65	585
Column definitions are given in the Appendix 1							Personnel time costs	33,660
							Non-personnel costs	
							Travel	360
							Training materials	250
							Assessment and evaluation materials	1500
							Recording equipment	1000
							Office supplies	600
							Software for case management	2000
							Total implementation costs	39,370

### Step 1: Name the implementation strategy and list the associated actions, actors, and temporality

In this first step of process mapping, the Proctor et al. framework is used to specify the procedures that comprise the implementation strategy. This includes the following:

1. Naming the implementation strategy in a way that is consistent with the existing guidelines and taxonomies in the literature [30, 36, 37].
2. Identifying and listing the strategy actions (i.e., what needs to be done) required for the successful execution of the implementation strategy.
3. Identifying the temporality of each action (when the action is performed; start and end dates of the actions).
4. Identifying and listing the actors (who will enact the actions) involved in each action.

The initial goal is to fully understand and document the implementation process and create a blueprint of the implementation strategy. This requires careful study of the implementation protocol and detailed operational information from the key personnel (e.g., project managers, facilitators) who are most familiar with the implementation project, to ensure comprehensiveness and accuracy. Key personnel directs the process-mapping component. They start by identifying the high-level implementation strategies and then drill down into the actions that occur in each strategy. An action is a discrete activity involving one or more resources—personnel and/or equipment. Developing a process map enables key personnel to describe all the main actions involved in delivering the implementation strategy along with the resources consumed at that action.

As shown in Table 1, we specified five discrete implementation strategies (column I): initial work for site readiness, initial training of the partners, ongoing administrative support, remote consultation, and fidelity review. A complete list of main actions is shown in column II, including activities such as holding in-person and videoconference meetings, making phone calls and exchanging emails, conducting an initial training workshop, conducting consultation and feedback sessions with clinicians via videoconferencing, and filling out fidelity assessment forms.

Column III describes temporality, or when each implementation strategy and its associated actions occur. Implementation efforts span a total of 24 months, including pre-implementation, implementation, and sustainment periods. For example, initial work for site readiness takes place during the first 3 months (pre-implementation), as does the training of the partners in a one-time workshop that lasts 2 days. Ongoing technical and administrative support, remote consultation, and fidelity review take place during the 12-month implementation period and the 6-month sustainment period. As noted in Table 1, temporality should include information on the specific start and end dates of the activities; this defines the observation period for which the frequency of the actions will be determined. The implementation actors (or personnel resources) are listed in column IV. In our case study, they include a team of one general practice facilitator, one expert liaison psychologist, one site administrator, and three site clinicians.

Implementation researchers can prepare a TDABC template prospectively by identifying implementation strategies, actions, temporality, actors, planned frequency, and unit duration of actions. Because implementation strategies are often standardized (e.g., procedure frequency and duration are decided beforehand) [38], such a TDABC template can be filled out a priori based on the study protocol and serve as a blueprint prior to the start of implementation.

When implementation efforts start, the TDABC template serves as a monitoring tool to track the real-time implementation process and can be used to track the strategy as delivered. The initial estimation of costs in the matrix can then be compared to the costs of the implementation strategies as actually delivered [8, 39–41] to compare estimated versus actual costs. Direct observation of the actions might reveal variations in the strategy as planned, allowing for documentation of required revisions in the initial template and yielding a map of the strategy as delivered in real time. This element of the method is consistent with calls within implementation science to determine how strategy delivery varies from what was originally intended and to assess the implications of this variation for implementation outcomes. The TDABC process map can be used as a monitoring tool for the delivery of implementation strategies.

### Step 2: Determine the frequency and average duration of each implementation action by actors, as well as actors’ total time spent on each action

Since information on activity frequency (i.e., how many times the activity occurs) and average duration (i.e., how long it takes to perform one unit of activity) is central to TDABC, process map actions need to be operationalized as concrete, observable, single events. For every action, a “unit of action” should be specified so that action frequency and average duration can be quantified. For example, a unit action can be delivering one training session. The average “unit duration” is then how long it takes to complete the unit action on average (e.g., the average duration of one training session). The total time spent on an action is determined by multiplying the observed action frequency within the timeline that the action takes place (temporality) and the action unit duration (the average time spent to perform one unit of action).

In Table 1, for each implementation strategy action, columns V and VI show its frequency and unit duration, respectively. Additional detail regarding how the action frequencies and unit durations are specified may be found in Appendix 2. For example, for the action “Provide feedback via videoconferencing,” the unit action is one single videoconference call. The frequency of action specifies how many videoconference calls take place, and the action unit duration is the average length of a single videoconference call. The values in column VII are derived by multiplying the action frequency by each actor (columns IV and V) and unit duration (column VI), yielding total time spent on the action by the specific actor.

### Step 3: Determine the price per hour of each actor

Information needed for calculating the price per hour of the resources comes from the project budget. Wage rate, or price per unit hour, is the total spending on the resource (e.g., salary) divided by the total hours provided by the resource.

In Table 1, column VII presents the price per actor (personnel) hour, which is calculated by dividing each person’s annual salary (including employee benefits) by 2080 annual work hours. In the case of consultant time, this would represent the hourly billing rate.

### Step 4: Determine non-personnel, fixed resources, and their associated expenses

Non-personnel resources whose costs are fixed and not time-driven (such as consumable equipment, supplies, and technology required to perform the actions) are itemized and the expense of each item is determined. This information usually can be obtained from the project budget.

In Table 1, non-personnel resources shared across actions include spending on items like training materials, assessment and evaluation materials, recording equipment, office supplies, and software for case management. These are fixed costs rather than time-driven, so we report their costs separately at the bottom of Table 1.

### Step 5: Calculate total costs

The total time spent on each action by each actor (step 2) is multiplied by the price per hour of the actor (step 3) in order to obtain the action cost for each actor. The sum of action costs yields the cost of personnel resources for the implementation strategy. Non-personnel, fixed costs are added to derive the total cost of the implementation strategy.

Finally, column IX in Table 1 shows the action costs. These are calculated by multiplying the total time spent on the action (column VII) by the price per hour (column VIII). Total personnel time costs, non-personnel expenses, and total cost of the implementation project are then summed and listed at the bottom of the table.

### Transforming the TDABC matrix into user-friendly reports and decision tools

**Fig. 1:**
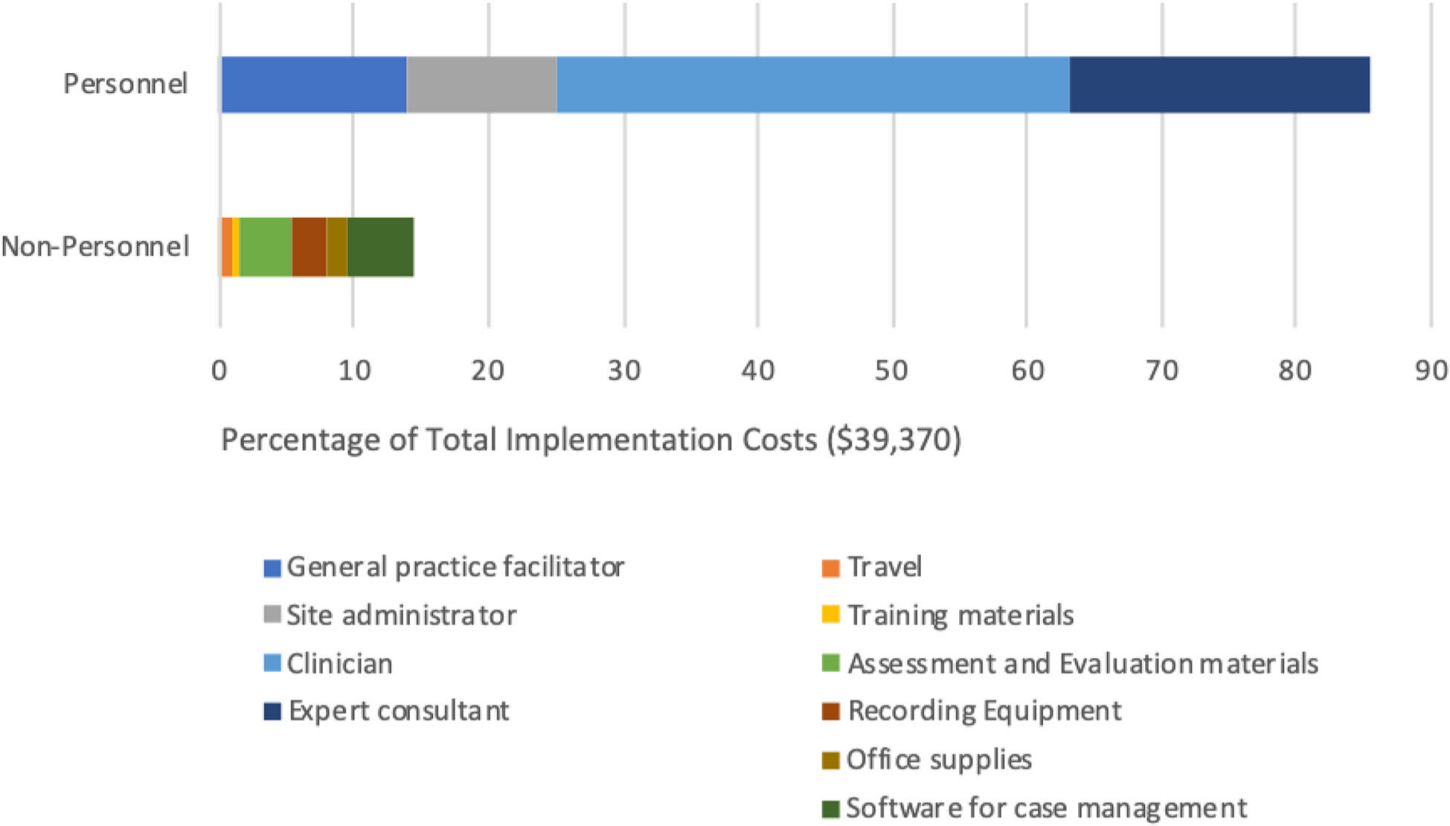
Composition of implementation costs

**Fig. 2:**
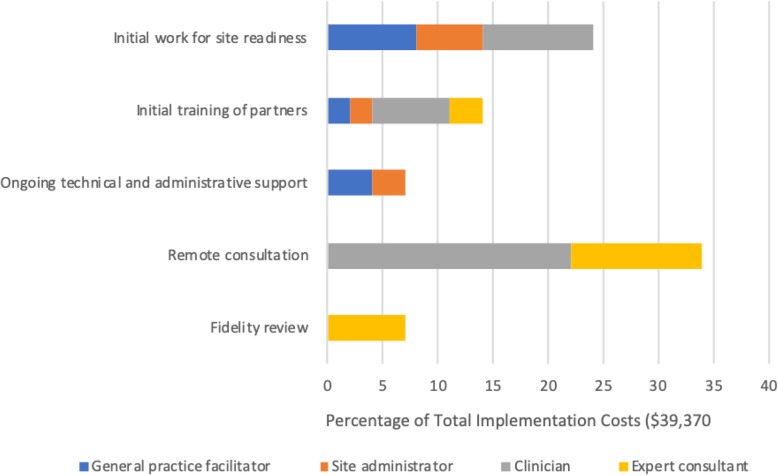
Cost composition by implementation strategy

**Fig. 3:**
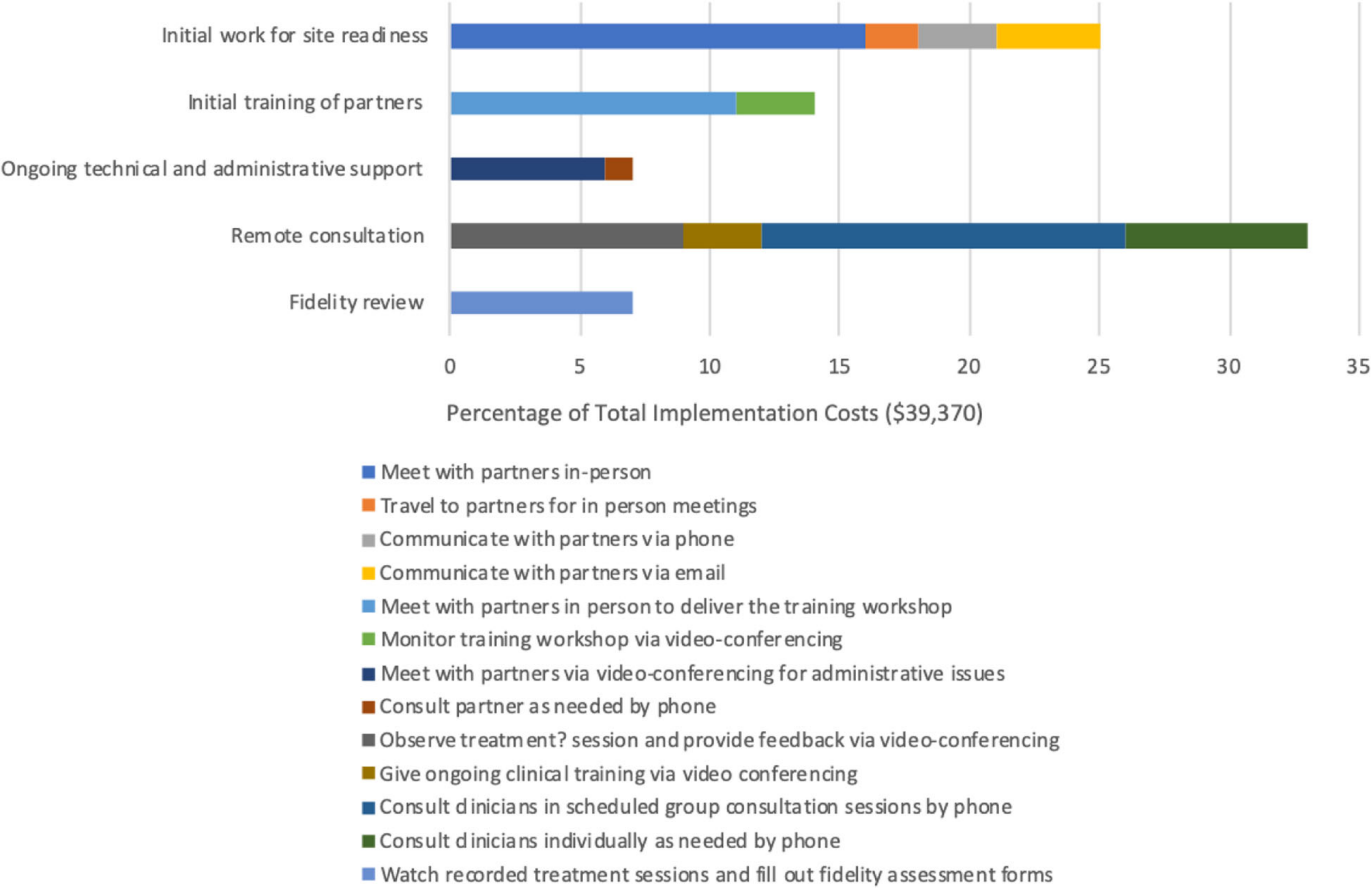
Action cost by implementation strategy

**Fig. 4:**
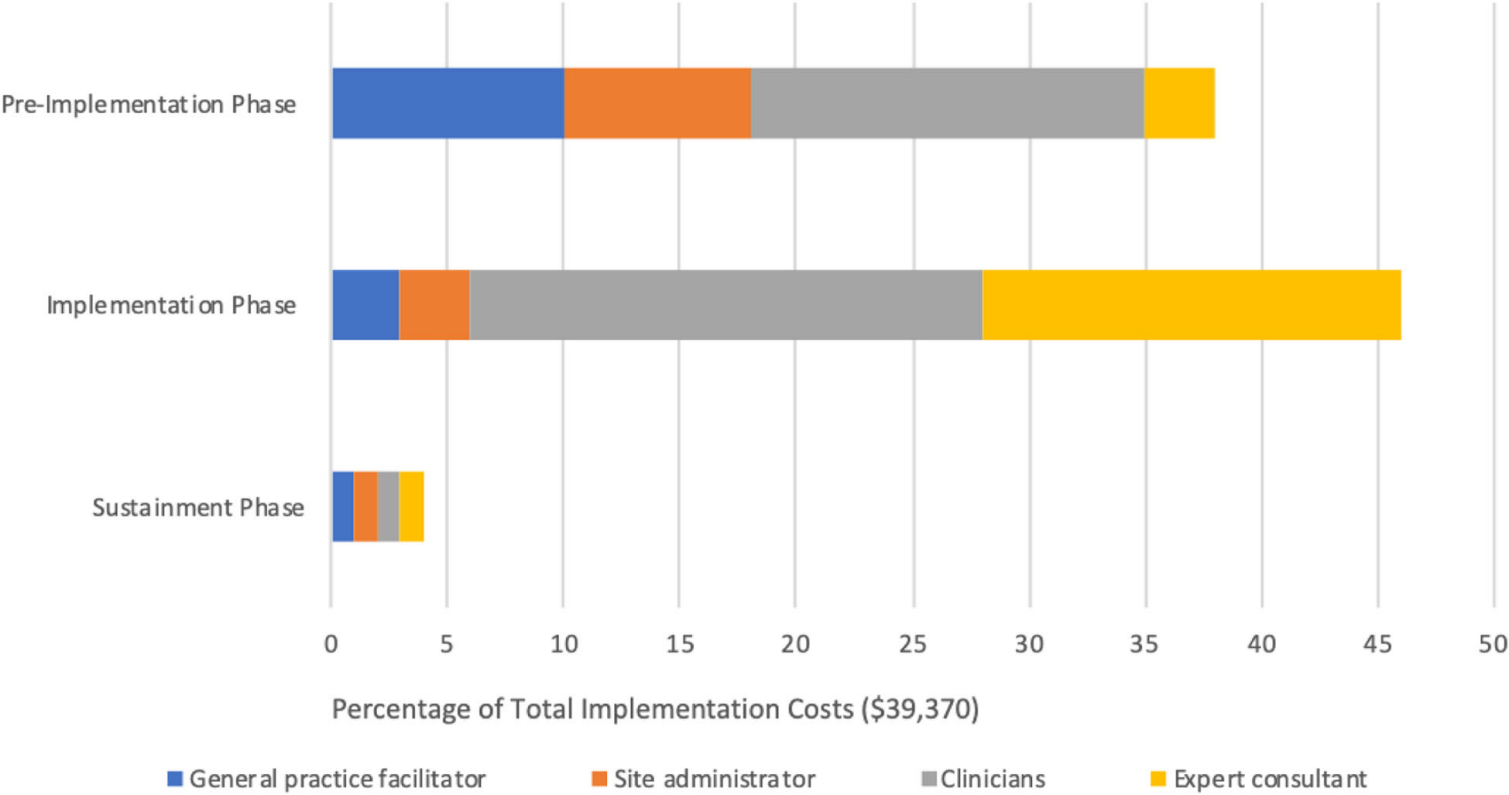
Cost composition by implementation phase

The detailed information captured in the final TDABC resources matrix (Table 1) can be transformed into numerous visual representations of project costs that can help stakeholders to understand both personnel and non-personnel resource allocation and associated costs. Figures 1, 2, 3, and 4 illustrate sample infographics that can be useful in assessing costs and making decisions. Figure 1 focuses on the composition of total costs (cost by personnel, the cost for each non-personnel item). Figure 2 illustrates the costs for each of the five implementation strategies used in our case study, including what portion of the costs of each strategy is devoted to each actor or personnel member. Figure 3 shows the cost of each specific action that is performed as part of each implementation strategy, and Fig. 4 shows costs by implementation phase, including the cost of specific personnel time within each phase.

These visuals illustrate how the proposed method produces data that provides concrete and transparent cost information for decision-makers. It can support the identification of actions with little value, causes of variation in costs, and how changing a specific component would change costs. Detailed resource composition enables decision-makers to look for the best balance among the resources allocated to the implementation strategies and to help identify places where it may be possible to reduce costs without substantially reducing effectiveness. For example, if it is apparent that travel time costs represent a large percentage of the overall cost burden, decision-makers may opt to use technology to conduct some meetings remotely rather than in person. Detailed information can also help to identify cases where it may be appropriate to shift specific tasks to less expensive personnel.

## Discussion

In this study, we present a simple, pragmatic method for costing implementation strategies that combines time-driven activity-based costing, an established business accounting method, with the Proctor et al. framework, a well-known implementation science framework for specifying and reporting implementation strategies [20, 21, 23]. Blending these two approaches allows for the integration of cost estimation into an existing structure that can guide evaluations of implementation strategy effectiveness. It involves clearly mapping implementation strategies by specifying the names, actions, actors, and temporality associated with them; determining the frequency and average duration of each action associated with individual strategies; and assigning a dollar value to the resources that each action consumes. Itemization of resource use and costs is piggy-backed on the Proctor framework; specifications of actions, actors, temporality, frequency, and average duration are used to directly determine the resources utilized by the strategies and their costs. Our approach allows researchers using the Proctor rubric to estimate implementation strategy costs and routinely report them alongside the other elements of the framework.

The detailed information produced by our approach benefits implementation strategy effectiveness research by providing a direct link between the implementation inputs (resources utilized) and implementation outcomes. Such information is especially useful for studying the mechanisms and processes that determine the effects of an implementation strategy on an implementation outcome, which is another core element in the Proctor et al. framework. For example, the effect of the implementation strategy dosage, reflected by action frequency and unit duration of the actions, is an important area of inquiry in implementation science [8, 23, 42]. Another benefit of our approach is that it offers an important opportunity to study site differences in the actual implementation process, such as when there is variation in the delivery of implementation strategies across implementation sites. A TDABC resource matrix can be created for each site to reflect site-specific information, and cross-site comparisons can be conducted to identify variations in resource use and costs, such as may occur when a site needs more support. This element of the method is consistent with calls within implementation science to determine how strategy delivery varies across sites and to assess the implications of this variation for implementation outcomes.

Although several implementation science frameworks include costs as an important implementation outcome to be studied alongside other implementation measures, in practice, cost estimation has not been integrated into most evaluations of implementation strategy effectiveness [5, 8]. The practicality of TDABC comes from its reliance on only two pieces of information that are often straightforward to determine: action frequency and average duration of unit action. In the context of implementation strategies, a majority of the relevant information can be obtained easily and accurately since the core activities that comprise an implementation strategy are typically specified in advance and then tracked and documented as part of implementation (e.g., documentation of consultation sessions for fidelity assessment) [38]. Thus, capturing this process data (e.g., number of consultation sessions, average duration of one consultation session) for costing purposes may not impose added burden. For standard, predictable or regular actions (e.g., weekly, 1-h stakeholder meetings; a 1-day training workshop), accurate data can be obtained easily if the actions are performed as planned. For more complex actions such as those of tailored implementation strategies, data can be obtained by direct observations as part of the process data. Applications of TDABC in organizational settings have demonstrated that determining activity frequencies and establishing an average duration is less burdensome than asking personnel to log their time and activities [15, 19, 21, 27]. The ability to observe these activities objectively likely makes documentation more accurate and reliable than personnel activity logs or general self-reported estimations of percentage effort. This is highly relevant in healthcare settings, where remembering to complete these logs may be especially challenging due to busy schedules and competing priorities.

As with all methods, this approach has limitations. Some activities, such as those requiring as-needed or ad hoc communication (e.g., phone calls, emails), are more difficult to capture in a standardized way and likely still require data collection via staff survey or logs, such as tracking communication frequency and average duration during a typical week during each implementation phase. Nonetheless, the majority of implementation activities are relatively straightforward to capture using this method (e.g., training workshops, scheduled consultation sessions, fidelity review sessions), thereby offering implementation science researchers an additional option for assessing costs via a systematic, detailed approach.

## Conclusion

Availability of a step-by-step procedure to guide and standardize the application of TDABC in the costing of implementation strategies, following an implementation science framework that is widely used, may encourage academic researchers to perform TDABC costing studies and build our knowledge base in this area with detailed and transparent cost information and quality cost evaluation. This framework also provides an approach for how to better evaluate and control implementation procedures and associated costs, which should facilitate better decision-making when choosing implementation strategies and associated logistics.

Future research should test this method in costing implementation strategies, especially in cases where strategy actions can be granularized to represent multiple levels and components of the strategy. Efforts to build a TDABC toolkit to track implementation procedures and resource use would be useful, especially because many implementation strategies are likely to share common or routine actions. Just like prior efforts to compile implementation strategies with specific labels and taxonomies, efforts to compile a list of common actions across strategies would be useful to help researchers map the actions involved in implementation strategies. This would enhance efforts to standardize and systematize the study of implementation strategy costs.

## Data Availability

Not applicable.

## Appendix A

**Table 2:** Sample costing matrix

	Actions	Temporality	Actors	Action frequency	Action unit duration	Total time spent	Wage rate	Total cost
Implementation strategy name	Action 1	Period action enacted Action start and end date	Actor A	#	Hours	Hours	$/hour	$
			Actor B	#	Hours	Hours	$/hour	$
	Action 2	Period action enacted Action start and end date	Actor C	#	Hours	Hours	$/hour	$
	Action 3	Period action enacted Action start and end date	Actor A	#	Hours	Hours	$/hour	$
			Actor B	#	Hours	Hours	$/hour	$

Implementation strategy name: label consistent with the existing guidelines and taxonomies in the literature

Actions: what needs to be done for the successful execution of the implementation strategy, listed as concrete, observable, quantifiable events

Temporality: timeline (start and end dates) of the action

Actors: who will enact the action

Action frequency: how many times the action is performed

Action unit duration: average duration of a single occurrence of the action

## Appendix B

**Table 3:** Determination of action frequency

Implementation strategy name	Actions	Action frequency
Initial work for site readiness	Meet with partners in person	# in-person meetings
	Travel to partners for in-person meetings	# in-person meetings that required a visit to partners
	Communicate with partners via phone	# of phone calls
	Communicate with partners via email	# emails processed
Initial training of partners	Meet with partners in person to deliver the training workshop	# workshop sessions
	Monitor training workshop via videoconferencing	# workshop sessions monitored by expert consultants
Ongoing administrative support	Meet with partners via videoconferencing for administrative issues	# videoconference meetings
	Consult partner as needed by phone	# phone calls
Longitudinal remote consultation	Observe EBP delivery sessions and provide feedback via videoconferencing	# conference calls
	Give ongoing clinical training via video conferencing	# video conference sessions for training purposes, individually to clinicians
	Consult clinicians in scheduled group consultation sessions by phone	# consultation sessions
	Consult clinicians individually as needed by phone	# phone calls
Fidelity review	Watch recorded EBP delivery sessions and fill out fidelity assessment forms	# of recorded sessions reviewed for fidelity
